# Demographic, social, psychological, mental health, and academic correlates of problematic smartphone use among French psychology students

**DOI:** 10.3389/fdgth.2026.1784927

**Published:** 2026-07-03

**Authors:** Germano Vera Cruz, Clarice Da Rosa, Yasser Khazaal

**Affiliations:** 1 Department of Psychology, UR 7273 CRP-CPO, University of Picardie Jules Verne, Amiens, France; 2 Addiction Medicine, Department of Psychiatry, Lausanne University Hospital and Lausanne University, Lausanne, Switzerland; 3 Research Centre, University Institute of Mental Health at Montreal and Department of Psychiatry and Addiction Montreal University, Montreal, QC, Canada

**Keywords:** problematic smartphone use, smartphone use frequency, behavioral addiction, social relationships in digital era, cognitive difficulties, impulsivity, self-regulation, future-self connection

## Abstract

**Background:**

Problematic smartphone use (PSU) among university students has raised increasing concern due to its links with psychological distress, cognitive difficulties, and academic impairment. Despite extensive international research, data on PSU among French university students—particularly psychology students—remain limited. Psychology students constitute a distinctive population because they receive formal training on addiction mechanisms, yet may still engage in maladaptive smartphone use, illustrating a potential intention–behavior gap.

**Objectives:**

This study aimed to (1) estimate the prevalence and severity of PSU among French psychology students; (2) examine sociodemographic, behavioral, social, psychological, mental health, and academic correlates and predictors of PSU score severity; and (3) test whether social variables moderate and mental health variables mediate the relationship between smartphone use frequency (SUF) and PSU severity.

**Methods:**

A cross-sectional online survey was conducted among 973 psychology undergraduate and master's students recruited from two French universities. Participants completed measures of PSU, smartphone use behaviors, social resources, cognitive functioning, impulsivity, personality traits, future-self connection, mental health, and academic functioning. Correlation analyses, multivariate linear regression, and moderation/mediation analyses with bootstrapping were performed.

**Results:**

The mean PSU score was slightly below the midpoint of the 1–7 scale. Using a conservative estimation method, the prevalence of PSU was between 1.64% and 5.14%. However, according to an alternative prevalence estimation method, 25% of participants exceeded the cut-off score of 4.14. Higher PSU scores was observed among younger students, women, bisexual participants, and undergraduates. Impulsivity emerged as the strongest predictor of PSU, followed by smartphone-related problems, SUF, and working memory difficulties. Lower social support, lower conscientiousness, higher neuroticism, poorer mental health, and greater academic-smartphone use interferences were associated with higher PSU. Sleep difficulties, depressive mood, and self-esteem partially mediated the SUF–PSU relationship. Community engagement and future-self connection unexpectedly strengthened, rather than buffered, the SUF–PSU association.

**Conclusions:**

PSU constitutes an issue of relevance among French psychology students despite high awareness of addiction-related risks. Findings highlight the central role of impulsivity, cognitive control, and mental health, and suggest that awareness alone is insufficient to prevent PSU. Interventions should focus on self-regulation, emotional coping, and context-specific academic smartphone use.

## 1 Introduction

Problematic smartphone use (PSU) refers to a pattern of excessive, poorly controlled smartphone engagement that leads to significant functional impairment and negative consequences in daily life (1, 2). Although PSU is not formally recognized as a behavioral addiction in major diagnostic classifications, it shares core features with addictive behaviors, including symptoms such as salience, loss of control and continued use despite harmful consequences (3, 4). Contemporary theoretical models conceptualize PSU as the result of an interaction between individual vulnerabilities (e.g., impulsivity, emotional dysregulation), situational factors, cue-reactivity, and reinforcing technological affordances via compensation and gratifications such as rewards from constant connectivity, notifications, and algorithmic personalization (5–7). It often includes preference for using smartphone rather than engaging in offline interpersonal or social activities (8).

### 1.1 Prevalence and determinants of PSU

While epidemiological studies frequently report PSU among adolescents and young adults, meta-analytic prevalence estimates range widely (approximately 10% to >30%). This substantial heterogeneity likely reflects methodological variability across studies—including differences in population characteristics, assessment instruments, and cutoff criteria—together with the absence of an agreed-upon framework for defining PSU (1, 9, 10). University students appear especially vulnerable due to developmental factors, academic demands, and pervasive reliance on smartphones for social, academic, and leisure activities. European studies, including those conducted in France, have reported PSU estimates among student populations in the range of 20%–35% with higher rates from samples of social sciences and psychology, however, these figures should be interpreted with caution, as they are highly sensitive to sample characteristics, assessment instruments, and the criteria used to define PSU (11–15). These findings highlight the importance of population-specific investigations using clearly defined thresholds.

A substantial body of literature has examined sociodemographic determinants of PSU. Younger age is consistently associated with higher PSU, reflecting developmental vulnerabilities related to self-regulation and identity formation (11, 16). Gender differences are frequently reported, with women often exhibiting higher PSU scores, potentially due to greater use of smartphones for social interaction and emotion regulation (17, 18). Sexual minority individuals have also been found to report higher levels of PSU, possibly reflecting greater reliance on digital spaces for social support and identity affirmation (19). Lower educational level, single relationship status, and lower income have likewise been associated with increased PSU, suggesting that socioeconomic and relational resources may act as protective factors (20).

Smartphone use behavior represents a proximal determinant of PSU. High smartphone use frequency (SUF), particularly for non-essential or entertainment-related activities, is one of the strongest predictors of PSU (1, 12, 21). Experiencing prior problems related to smartphone use, perceiving a need to reduce use, and engaging in compensatory behaviors such as installing apps or using embedded tools to control usage have all been associated with higher PSU, likely reflecting needs and attempts to improve self-regulation rather than effective control (5, 21, 22). These variables may therefore serve as behavioral markers of PSU severity.

Psychological vulnerabilities play a central role in contemporary models of PSU. Cognitive difficulties, particularly deficits in working memory and attentional control, have been associated with increased distractibility and compulsive smartphone checking (23–25). Impulsivity is one of the most robust psychological predictors of PSU, facilitating immediate gratification seeking and undermining long-term self-control (26–28). Personality traits are also relevant: conscientiousness is generally negatively associated with PSU, whereas neuroticism shows consistent positive associations (29). Emerging evidence further suggests that a weaker sense of connection with one's future-self may contribute to addictive behavior by reducing the salience of long-term consequences (30–32).

PSU has been repeatedly linked to poorer mental health outcomes. Sleep difficulties, including prolonged sleep onset latency and nocturnal awakenings, are among the most consistently reported correlates, partly due to bedtime smartphone use and cognitive hyperarousal (33, 34). Depressive symptoms and lower self-esteem are also strongly associated with PSU, with bidirectional relationships suggested by longitudinal studies (35, 36). Smartphones may function as tools for mood regulation, but excessive reliance can exacerbate emotional distress, creating a self-reinforcing cycle of use and psychological vulnerability.

In academic contexts, PSU has been associated with reduced academic performance and engagement. Smartphone-related multitasking during lectures or study time interferes with attention, working memory, and learning consolidation (37–39). Higher PSU has been linked to lower grade point averages and increased academic procrastination, even after controlling for general study habits (40).

### 1.2 Rationale, objectives, and theoretical ground of the present study

#### 1.2.1 Rationale for the present study

Despite growing international literature, data on PSU prevalence and its multidimensional determinants among French university students—especially psychology students—remain limited. Moreover, few studies have simultaneously examined sociodemographic, behavioral, social, psychological, mental health, and academic variables within an integrated analytical framework, while also testing mediation and moderation mechanisms.

Psychology students are a distinctive population for studying problematic smartphone use (PSU) because they receive formal training on addiction mechanisms and risks. Despite higher awareness and knowledge, this might not necessarily translate into healthier behaviors, illustrating the intention–behavior gap (41). Their academic context may increase vulnerability to PSU through emotional demands, stress, and frequent reliance on smartphones for learning, information seeking, and emotion regulation. Smartphones may thus serve compensatory coping functions even among well-informed individuals (5). In this context, it seems important to test whether social factors moderate, and mental health factors mediate, the relationship between smartphone use and PSU (42). Findings can inform targeted prevention strategies and improve training for future mental health professionals.

#### 1.2.2 Objectives of the present study

Thus, the present study aims at (a) estimating the prevalence and severity of PSU among French psychology university students; (b) examining correlational and predictive relationships between PSU severity and a broad set of sociodemographic, smartphone use behavior, social, psychological, mental health, and academic variables; and (c) testing whether social variables moderate, and mental health variables mediate, the relationship between smartphone use frequency and PSU. By adopting this comprehensive approach, the study seeks to contribute to a more nuanced understanding of PSU and inform targeted prevention and intervention strategies in university settings.

#### 1.2.3 Theoretical ground of the study

First, as noted at the beginning of this introduction, the assessment of problematic smartphone use (PSU) remains a subject of ongoing debate, and prevalence estimates vary considerably depending on the measurement instrument, conceptual framework, and cut-off criteria employed. This variability reflects broader challenges in the operationalization of PSU rather than limitations associated with any particular instrument (3, 43, 44). In the present study, PSU was assessed using the Smartphone Application-Based Addiction Scale (SABAS) (45). This instrument was developed based on Griffiths' components model of addiction (46), which conceptualizes behavioral addictions through six core dimensions: salience, mood modification, tolerance, withdrawal, conflict, and relapse. Rather than focusing on specific smartphone activities or overall usage frequency, the SABAS assesses addiction-like symptoms associated with smartphone application use. This theoretically grounded approach enables researchers to investigate problematic smartphone-related behaviors in a parsimonious and conceptually coherent manner. Importantly, the SABAS has demonstrated satisfactory psychometric properties across diverse cultural contexts, including evidence of reliability, construct validity, and measurement invariance (1, 45, 47, 48). Furthermore, its widespread adoption in international research facilitates meaningful comparisons across studies and populations. More importantly, the primary objective of the present study was not to estimate a universal prevalence rate of PSU but rather to determine its prevalence within the specific study population and to examine its associations with relevant psychological and sociodemographic variables. In this context, the principal requirement is a measure capable of reliably and validly capturing individual differences in PSU, and the SABAS has consistently demonstrated such properties.

Second, the selection of variables and the formulation of hypotheses were informed by both the empirical literature reviewed in this introduction and contemporary theoretical models of problematic smartphone use and behavioral addictions. In particular, the study was guided by: (a) the Interaction of Person–Affect–Cognition–Execution (I-PACE) model (4, 7), which posits that internet-use disorders emerge from dynamic interactions among individual predispositions (e.g., personality traits and psychopathological vulnerabilities), affective and cognitive responses to internal and external cues, and executive-control processes; through repeated reinforcement, these interactions may foster maladaptive behaviors that progressively evolve into loss of control and addictive use patterns; (b) compensatory internet use theories (42), which emphasize the use of digital technologies as a means of coping with unmet psychological needs or negative emotional states; and (c) theoretical frameworks highlighting the role of impulsivity, deficits in cognitive control, emotional vulnerabilities, and social resources in the development and maintenance of PSU (3, 5). Together, these perspectives provide a coherent theoretical foundation for examining the multifaceted psychological, cognitive, emotional, and social factors associated with problematic smartphone use.

### 1.3 Research questions and hypotheses

Four research questions (RQs) guided the present study. **RQ1** examined the mean level of problematic smartphone use (PSU) in the sample and the proportion of participants meeting the proposed diagnostic threshold for PSU measured with the SABAS (45). Peng et al. (49), employing latent profile analysis and ROC-based sensitivity analysis, proposed a threshold of 23 out of 36 on the SABAS (1–6 scale), equivalent a total score of 29 out of 42 when employing a 7-point scale, corresponding to a mean score of 4.14 out of 7. Using this criterion, 18.9% of 63,205 Chinese adolescents were classified as “high-risk” users, yielding an overall PSU prevalence of 21.1%. Rochat et al. (1) recommended a more conservative approach to estimate PSU as excessive use impairing functioning: participants scoring >4.5 on the 1–6 SABAS scale and at least one of the following conditions: a response of “agree” or “strongly agree” on the conflict item of the SABAS (“Conflicts have arisen between me and my family/friends because of my smartphone use”) and a response of “definitely yes” on the unique item in the survey directly querying functional impairment (“In the last year, has your use of the smartphone caused you problems in your personal, professional or school/university life?”). Applying this method to a representative U.S. adult sample (*n* = 1,989), 13% (*n* = 267) scored >4.5, of whom 1.2% (*n* = 24) reported conflicts and 0.75% (*n* = 15) reported functional impairment. **RQ2** investigated the extent to which the modeled sociodemographic, smartphone use–related, social, psychological, mental health, and academic variables were correlated with participants' PSU scores. **RQ3** assessed the extent to which these same sets of variables predicted PSU scores. **RQ4** examined whether selected social, psychological, and mental health variables moderated and/or mediated the relationship between smartphone use frequency (SUF) and PSU.

The sociodemographic variables included age, gender, sexual orientation, relationship status, education level, and income. Based on prior research, we hypothesized that younger (vs. older), female (vs. male), bisexual or homosexual orientation (vs. heterosexual orientation), single (vs. in a relationship), undergraduate (vs. master's-level) students, and students with lower (vs. higher) income would report significantly higher PSU levels.

Smartphone use–related variables included SUF for non-essential activities, having experienced problems related to smartphone use, use of smartphone applications to improve well-being, perceived need to reduce SUF, and use of embedded smartphone tools to control SUF. We hypothesized that higher levels of these variables (or affirmative vs. negative responses) would be associated with higher PSU scores.

Social variables comprised the number of leisure outings with others, the number of close relationships engaged in leisure activities, satisfaction with family and friend relationships, satisfaction with intimate relationships, perceived social support, level of community engagement (e.g., involvement in non-profit or community organizations), and level of religious engagement. We hypothesized that higher levels of these social resources would be associated with lower PSU.

Psychological variables included working memory–related cognitive difficulties, impulsivity, personality traits, and perceived connection with the future-self. We hypothesized that greater cognitive dysfunction and impulsivity would be associated with higher PSU, whereas conscientiousness would be negatively associated with PSU scores and neuroticism positively associated. Stronger connection with the future-self was expected to be associated with lower PSU.

Mental health variables included sleep difficulties, depressive mood (vs. happiness), and self-esteem. We hypothesized that greater sleep problems, higher depressive mood, and lower self-esteem would be associated with higher PSU.

Academic variables included smartphone-related interference with university work (multitasking during academic activities) and mean academic grade from the previous semester. We hypothesized that greater academic interference would be associated with higher PSU, whereas higher academic performance would be associated with lower PSU.

Finally, we hypothesized that social variables would moderate the relationship between SUF and PSU, and that mental health variables would partially and significantly mediate the relationship between SUF and PSU.

## 2 Methods

### 2.1 Inclusion criteria

The inclusion criteria for the study were: being 18 years of age or older; being enrolled as an undergraduate or master's degree student in psychology; being registered at and attending courses in a French university (or an equivalent higher education institution); and agreeing to participate voluntarily by providing informed consent.

### 2.2 Participants

A total of 1,002 students from two French universities completed the online survey questionnaire. However, 29 participants were excluded for failing the attention-check items embedded in the questionnaire (see the Data Analyses subsection for details). The final sample therefore comprised 973 participants.

Participants ranged in age from 18 to 43 years, although the 18–25 age group was overrepresented (18–21 years: *n* = 853, 87.66%; 22–25 years: *n* = 104, 10.69%; 26 years and older: *n* = 16, 1.64%). Female participants largely outnumbered male participants (female: *n* = 660, 67.83%; male: *n* = 303, 31.14%; other: *n* = 10, 1.03%). This gender imbalance is consistent with national statistics indicating that women are substantially more likely to pursue studies in the humanities in French universities [70.9% of students in 2023 (50)]. Regarding educational level, 761 participants (78.21%) were undergraduate students and 212 (21.70%) were enrolled in a master's degree program. Detailed sociodemographic characteristics are presented in Table 1.

**Table 1:** Descriptive statistics of all variables included in the study.

Variable/measures	Scale/min–max	Mean	SD	Skewness*	Kurtosis*	Frequency
Sociodemographics Variables						
Age	18–43	19.97	2.211	4.132	27.206	18–21 years-old = 853 (87.66%)
						22–25 years-old = 104 (10.69%)
						26 + years-old = 16 (1.64%)
Gender	–	–	–	–	–	Male = 303 (31.14%)
						Female = 660 (67.83%)
						Other = 10 (1.03%)
Sexual orientation	–	–	–	–	–	Heterosexual = 751 (77.18%)
						Bisexual = 145 (14.90%)
						Homosexual = 47 (4.83%)
						Other = 30 (3.08%)
Relationship status	1–5	–	–	–	–	Single with no sexual partner = 432 (44.40%)
						Single with occasional sexual partner(s) = 72 (7.40%)
						Single with regular sexual partner(s) = 45 (4.62%)
						In a monogamous relationship for several weeks/months = 140 (14.39%)
						In a monogamous relationship for more than a year = 284 (29.19%)
Education level	–	–	–	–	–	Undergrad = 761 (78.21%)
						Master's degree = 212 (21.70%)
Income	1–4	1.26	0.543	2.27	6.45	1 (income between 0 and 500 euros per month) = 757 (77.71%)
						2 (income between 500 and 1000 euros per month) = 187 (19.22%)
						3 (income between 500 and 1000 euros per month) = 19 (1.95%)
						4 (income of more than 1500 euros per month) = 10 (1.03%)
Smartphone use behavior measures						
Smartphone use frequency for non-essential activities (in last 3 months; measured in hours)	1–6	3.54	1.194	0.390	−0.545	
Having experienced personal, professional, or academic problems due to smartphone use (previous three months)	1–4	1.80	0.857	0.773	−0.277	
Use of smartphone applications to improve well-being (previous 12 months)	–	–	–	–	–	No = 552 (56.73%)
						Yes = 421 (43.27%)
Perceived need to control smartphone use frequency (previous 12 months)	–	–	–	–	–	No = 262 (26.93%)
						Yes = 711 (73.07%)
Use of embedded smartphone tools to control smartphone use frequency (previous 12 months)	–	–	–	–	–	No = 268 (27.54).
						Yes = 705 (72.46)
Smarphone problematic use measure						
SABAS salience	1–7	2.97	1.549	0.434	−0.616	
SABAS conflict	1–7	2.17	1.461	1.241	0.784	
SABAS mood modification	1–7	4.43	1.733	−0.515	−0.682	
SABAS tolerance	1–7	3.78	1.663	0.175	−0.953	
SABAS withdrawal	1–7	3.15	1.714	0.488	−0.735	
SABAS_relapse	1–7	3.54	1.755	0.267	−0.983	
SABAS total mean score	1–7	3.34	1.112	0.248	−0.410	
Social activities, relationships, and social engagement measures						
Number of leisure or entertainment outings with others (previous 30 days)	1–6	3.36	1.247	0.538	−0.406	
Number of close relationships (family members and friends) with whom participants engaged in leisure activities (previous 30 days)	0–27	10.20	6.04	0.662	−0.569	
Satisfaction (level of) with family members and friends relationship	1–5	3.77	0.956	−0.494	−0.168	
Satisfaction (level of) with intimate relationships	1–5	3.43	1.364	−0.453	−1.030	
Perceived (level of) social support from family and friends when in need	1–7	4.79	1.697	−0.296	−0.933	
Community (level of) engagement	1–7	2.71	1.697	0.775	−0.396	
Religious (level of) engagement	1–7	2.38	1.756	0.920	−0.484	
Psychological Measures						
WMQ working memory (short-term storage) (item1)	1–5	2.68	1.129	0.537	−0.522	
WMQ working memory (short-term storage) (item2)		2.76	1.243	0.458	−0.845	
WMQ working memory (short-term storage) (item3)		1.73	0.905	1.461	2.169	
WMQ working memory (short-term storage) total mean score	1–5	2.39	0.776	0.587	0.145	
WMQ attention (item4)	1–5	2.49	1.047	0.649	−0.179	
WMQ attention (item5)	1–5	2.72	1.103	0.514	−0.487	
WMQ attention total mean score	1–5	2.61	0.923	0.623	−0.254	
WMQ executive control (item6)	1–5	2.67	1.121	0.423	−0.601	
WMQ executive control (item7)	1–5	2.49	1.295	0.578	−0.745	
WMQ executive control (item8)	1–5	2.19	1.162	0.823	−0.172	
WMQ executive control total mean score	1–5	2.45	0.959	0.552	−0.323	
WMQ total mean score	1–5	2.47	0.737	0.556	0.131	
UPPS-P positive urgency (item1)	1–4	2.22	0.877	0.206	−0.727	
UPPS-P positive urgency (item2)	1–4	2.24	0.917	0.300	−0.729	
UPPS-P positive urgency (item3)	1–4	2.16	0.869	0.242	−0.731	
UPPS-P positive urgency (item4)	1–4	2.35	0.924	0.093	−0.861	
UPPS-P positive urgency total mean score	1–4	2.24	0.701	0.103	−0.495	
UPPS-P negative urgency (item5)	1–4	2.34	0.900	0.130	−0.770	
UPPS-P negative urgency (item6)	1–4	2.53	0.884	−0.091	−0.710	
UPPS-P negative urgency (item7)	1–4	2.13	0.886	0.351	−0.661	
UPPS-P negative urgency (item8)	1–4	2.19	0.886	0.314	−0.642	
UPPS-P negative urgency total mean score	1–4	2.29	0.682	0.217	−0.366	
UPPS-P urgency impulsivity total mean score	1–4	2.16	0.608	0.230	−0.287	
BFI-10 extraversion (item1)	1–5	2.68	1.186	.214	−0.867	
BFI-10 extraversion (item2)	1–5	2.65	1.172	0.337	−0.698	
BFI-10 extraversion total mean score	1–5	2.66	1.074	0.272	−0.719	
BFI-10 agreeableness (item3)	1–5	3.59	1.075	−0.522	−0.428	
BFI-10 agreeableness (item4)	1–5	2.97	1.215	0.050	−0.945	
BFI-10 agreeableness total mean score	1–5	3.27	0.862	−0.238	−0.361	
BFI consciousness (item5)	1–5	3.37	1.044	−0.199	−0.557	
BFI-10 consciousness (item6)	1–5	2.90	1.184	0.029	−0.868	
BFI consciousness total mean score	1–5	3.13	0.966	−0.072	−0.574	
BFI-10 neuroticism (item7)	1–5	3.54	1.364	−0.536	−0.952	
BFI-10 neuroticism (item8)	1–5	3.53	1.428	−0.516	−1.104	
BFI-10 neuroticism total mean score	1–5	3.53	1.298	−0.526	−0.926	
BFI-10 openness (item9)	1–5	3.37	1.256	−0.210	−1.050	
BFI-10 openness (item10)	1–5	3.71	1.295	−0.748	−0.563	
BFI-10 openness total mean score	1–5	3.71	1.295	−0.748	−0.563	
Connection (level of) with future-self 20 years from the present	1–11	6.25	2.887	−0.037	−0.841	
Mental health measures						
Sleep duration (from falling asleep until finally waking up; in last 6 months; self-estimation measured in hours)	3–9.70	7.92	1.360	−1.620	2.910	
Sleep difficulties (difficulty falling asleep and/or frequent waking up during the night; experienced in last 6 months)	1–5	3.52	1.109	−0.288	−0.894	
SDHS happiness (item1, reverse-coded)		2.78	0.854	−0.267	−0.566	
SDHS depression (item2)	1–4	1.71	0.736	0.673	−0.338	
SDHS depression (item3)	1–4	2.99	0.821	−0.505	−0.270	
SDHS happiness (item4, reverse-coded)	1–4	2.09	0.833	0.308	−0.603	
SDHS5 happiness (item5, reverse-coded)	1–4	1.88	0.775	0.501	−0.373	
SDHS depression (item6)	1–4	2.35	1.022	0.159	−1.105	
SDHS total mean score	1–4	2.30	0.577	0.206	−0.441	
SISE self-esteem	1–4	2.28	0.881	0.185	−0.701	
Academic measures						
MPIL-OC listening to music	1–11	7.77	3.571	−0.754	−0.871	
MPIL-OC writing/reading SMS	1–11	7.70	3.158	−0.626	−0.848	
MPIL-OC use social media	1–11	5.64	3.071	0.110	−0.987	
MPIL-OC speaking with others about on-line content	1–11	6.17	3.307	−0.059	−1.207	
MPIL-OC internet surf	1–11	7.39	2.970	−0.529	−0.755	
MPIL-OC watch video	1–11	6.05	3.564	−0.083	−1.388	
MPIL-OC writing/reading emails	1–11	5.52	3.362	0.255	−1.218	
MPIL-OC listening/sending voice messages	1–11	5.82	3.486	0.070	−1.341	
MPIL-OC playing video-games	1–11	3.50	3.004	1.097	0.037	
MPIL-OC online shopping	1–11	3.74	3.204	0.951	−0.408	
MPIL-OC chatting on phone	1–11	5.38	3.588	0.290	−1.341	
MPIL-OC attending on-line conferences	1–11	3.11	3.144	1.395	0.587	
MPIL-OC total mean score	1–11	5.64	2.326	−0.086	−0.674	
MPIL-IC listening to music	1–11	2.99	3.615	1.536	0.603	
MPIL-IC writing/reading SMS	1–11	6.58	3.176	−0.107	−1.126	
MPIL-IC use social media	1–11	4.82	3.175	0.456	−0.945	
MPIL-IC speaking with others about on-line content	1–11	5.75	3.352	0.100	−1.246	
MPIL-IC internet surf	1–11	6.04	3.162	0.044	−1.158	
MPIL-IC watch video	1–11	3.28	3.294	1.262	0.160	
MPIL-IC writing/reading emails	1–11	3.80	2.616	1.377	1.074	
MPIL-IC listening/sending voice messages	1–11	2.98	3.169	1.561	1.043	
MPIL-IC playing video-games	1–11	3.11	2.873	1.320	0.612	
MPIL-IC online shopping	1–11	2.79	2.816	1.572	1.362	
MPIL-IC chatting on phone	1–11	2.66	3.123	1.740	1.519	
MPIL-IC attending on-line conferences	1–11	1.96	2.351	2.637	6.043	
MPIL-IC total mean score	1–11	3.89	2.198	1.073	0.575	
Grade mean for the last university semester	1–6	3.87	0.994	0.310	−0.327	1 (grade between 0 and 5) = 2 (0.21%)
						2 (grade between 6 and 9) = 34 (3.49%)
						3 (grade between 10 and 11) = 400 (41.11%)
						4 (grade between 12 and 13) = 224 (23.02%)
						5 (grade between 14 and 16) = 283 (29.09%)
						6 (grade between 17 and 20) = 30(2.99%)

–Nominal variable (non-ordinal).

* A general guideline for skewness is that if the number is greater than +1 or lower than –1, this is an indication of a substantially skewed distribution. For kurtosis, the general guideline is that if the number is greater than +1, the distribution is too peaked.

SABAS  , Smartphone Application-Based Addiction Scale; BSMAS, Bergen Social Media Addiction Scale; MPIL (OC = interference with outside classroom university work; IC = interference during inside classroom lectures and/or seminars), Mobil Phone Interference in Life Scale; SDHS, Short Happiness-Depression Scale; WMQ, Working Memory Questionnaire; UPPS-P, Short version (20-item) Urgency-Premeditation; Perseverance-Sensation Seeking-Positive Urgency Impulsive Behavior Scale; BFI-10, 10-item Big Five Inventory.

There is a high likelihood that the sample is representative of the target population, as at least 75% of eligible students in the two participating universities completed the online survey.

### 2.3 Recruitment and procedures

Potential participants were approached during university classes. A professor or a trained research assistant presented the study objectives and informed students that participation was voluntary, fully anonymous, and without any negative consequences in the event of non-participation. Students who agreed to participate were provided with a link to the online survey, where they electronically signed the informed consent form before completing the questionnaire.

### 2.4 Ethics

Participants gave digital informed consent for participating in the study and completing the survey. Participation was voluntary and restricted to those aged ≥18. All data was anonymously collected. The study was conducted in accordance with Declaration of Helsinki on Ethical Principles for Medical Research Involving Human Subjects (51). Ethical approval for the research project (Decision n° 2026-95) was obtained from the Ethics Committee of the University of Picardie Jules Verne.

### 2.5 Measures

#### 2.5.1 Sociodemographic variables

Sociodemographic variables included age; gender (male, female, other); sexual orientation (heterosexual, bisexual, homosexual, other); relationship status [5-point scale: single with no sexual partner; single with occasional sexual partner(s); single with regular sexual partner(s); in a monogamous relationship for several weeks/months; in a monogamous relationship for more than one year]; education level (undergraduate student, master's degree student); and income (4-point scale: less than €500 per month; €500–1,000 per month; €1,000–1,500 per month; more than €1,500 per month).

#### 2.5.2 Smartphone use behavior variables

Smartphone use behavior variables included: smartphone use frequency (SUF) for non-essential activities during the previous three months (6-point scale: <1; 1–3; 3–5; 5–7; 7–9; >9 h); having experienced personal, professional, or academic problems due to smartphone use during the previous three months (4-point scale: never, rarely, sometimes, often); use of smartphone applications (apps) to improve well-being during the previous 12 months (no/yes); perceived need to control SUF during the previous 12 months (no/yes); and use of embedded smartphone tools to control SUF during the previous 12 months (no/yes).

Problematic smartphone use (PSU) was assessed using a French translation of the SABAS (45). The SABAS consists of six items [e.g., “Conflicts have arisen between me and my family (or friends) because of my smartphone use”]. Responses are rated on a 7-point Likert scale ranging from *Never* (1) to *All the time* (7). In the present study, internal consistency was acceptable (Cronbach's alpha [*α*] = .76).

#### 2.5.3 Social variables

Social variables included: (a) number of leisure or entertainment outings with others during the previous three months; (b) number of close relationships (family members and friends) with whom participants engaged in leisure activities during the previous three months; (c) satisfaction with family and friend relationships during the previous three months; (d) satisfaction with intimate relationships during the previous three months; and (e) perceived social support from family and friends when in need during the previous three months (all measured on 5-point Likert scales). In addition, (f) community engagement (e.g., involvement in non-profit or community organizations) and (g) religious engagement were assessed using 7-point Likert scales. Each social variable was measured using a single item.

#### 2.5.4 Psychological variables

Psychological variables included cognitive functioning difficulties, impulsivity, personality traits, and connection with the future-self. Cognitive difficulties related to working memory (short-term storage, attention, and executive control) were measured using a French translation and adaptation of eight items from the Working Memory Questionnaire [WMQ (52)]. Responses were rated on a 5-point Likert scale ranging from *Never* (1) to *Always* (5). Cronbach's *α* values for the three dimensions were .50, .64, and .72, respectively, with an overall α of .81.

Impulsivity was assessed using eight items corresponding to the positive and negative urgency dimensions of the UPPS-P Impulsive Behavior Scale (53). Items were rated on a 4-point Likert scale ranging from *Strongly disagree* (1) to *Strongly agree* (4). Internal consistency was satisfactory (*α* = .79 and .77 for the two dimensions; overall *α* = .83).

Personality traits were assessed using the French version of the 10-item Big Five Inventory (54), measuring extraversion, agreeableness, conscientiousness, neuroticism, and openness to experience. Items were rated on a 5-point Likert scale ranging from *Strongly disagree* (1) to *Strongly agree* (5). Cronbach's *α* values were .80, .23 (inter-item correlation *r* = .131, *p* < .001), .68, .84, and .27 (inter-item correlation *r* = .158, *p* < .001), respectively.

Connection with the future-self (55) was assessed using a single item: “To what extent do you feel connected to or concerned about your future-self (the person you will become) 20 years from today?” Responses ranged from *Not at all connected/concerned* (1) to *Very much connected/concerned* (11).

#### 2.5.5 Mental health variables

Mental health variables included sleep difficulties, happiness–depressive mood, and self-esteem. Sleep difficulties were assessed using a single item from the French version of the Sleep Health Index (56): “Over the past six months, have you had difficulty falling asleep or woken up frequently during the night?” Responses ranged from *Never* (1) to *Very often* (5).

Happiness–depressive mood was measured using the French translation of the Short Depression–Happiness Scale [SDHS (57)], a six-item instrument assessing happiness and depression. Items were rated on a 4-point Likert scale ranging from *Never* (1) to *Often* (4). Happiness items were reverse-coded so that higher scores reflected higher depressive mood. Cronbach's *α* values were. 77 (happiness) and .66 (depression), with an overall *α* of .77.

Self-esteem was assessed using the French version of the Single-Item Self-Esteem Scale [SISE (58)]. Responses ranged from *Very true for me* (1) to *Not at all true for me* (4).

#### 2.5.6 Academic variables

Academic variables included smartphone-related interference with university work (multitasking) and mean academic grade from the previous semester. Smartphone interference was measured using a French translation of the Mobile Phone Interference in Life questionnaire [MPIL (59)]. Participants reported the frequency of engaging in 12 smartphone-based activities (e.g., messaging, social media use, video viewing, gaming) during academic tasks using an 11-point Likert scale (*Never* to *Very often*). Two composite scores were calculated: interference during activities outside the classroom (*α* = .91) and interference during lectures or seminars (*α* = .91).

Mean academic grades from the previous semester were converted into six ordinal categories (0–5; 6–9; 10–11; 12–13; 14–16; 17–20), consistent with the French university grading system (0–20 scale).

### 2.6 Statistical analyses

The total PSU score was used as the dependent (outcome) variable. All other variables served as independent (predictor) variables.

Data quality was first assessed by identifying participants who failed the embedded attention-check items (e.g., “If you are reading this item, please select the… response”). Twenty-nine participants failed these checks and were excluded. No missing data or significant outliers were identified. Consequently, analyses were conducted on a final sample of 973 participants.

To address RQ1, descriptive statistics (means [*M*], standard deviations [SD], frequencies, skewness, kurtosis) and ANOVA analyses were conducted to examine PSU levels across sociodemographic groups. Descriptive analyses were also performed for all study variables.

To address RQ2, univariate correlation analyses were conducted between each independent variable and PSU. Pearson correlations were used for ordinal or continuous variables, and point-biserial correlations were applied for nominal or dichotomous variables, as recommended by Demirtas and Hedeker (60).

To address RQ3, a multivariate linear regression model was constructed, including only those predictors that were significantly associated with PSU in the univariate analyses. Univariate correlations assess bivariate associations without accounting for confounding variables, whereas multivariate regression estimates the unique contribution of each predictor while controlling for others (61, 62).

To address RQ4, mediation and moderation analyses were conducted using bootstrapping procedures (1,000 resamples) to obtain robust confidence intervals for indirect effects. SUF was specified as the predictor, PSU as the outcome variable, social variables and connection with the future-self as moderators, and mental health variables (sleep difficulties, happiness–depressive mood, and self-esteem) as mediators.

Descriptive analyses were performed using IBM SPSS Statistics [version 31.0 (63)]. All correlational, regression, mediation, and moderation analyses were conducted using Jamovi [version 2.4 (64)].

## 3 Results

### 3.1 The level of PSU among participants (answer to RQ1)

Table 1 presents de descriptive statistics related to all the study variables.

As shown on Table 1, the participants' PSU total mean score was slight below the mid-point of the scale [*M* = 3.34 (SD = 1.11), in a 1-7 scale, the mid-point being 3.5]. However, detailed descriptive statistics show the following distribution of the PSU total mean score: min = 1.50, max = 6.33; 390(40%) participants had *M* > 3.5; 247(25.3%) participants had *M* > 4 (more precisely 25.3% of participants had a score ≥ 4.16); 131(13.4%) participants had *M* > 4.5; 74(7.5%) participants had *M* > 5; 26(2.6%) participants had *M* > 5.5; 7(0.7%) participants had *M* > 6. It is important to note that, from the perspective of Peng et al. (49) suggested SABAS score cut-off, the current study PSU prevalence was 25.3%. However, from the perspective of Rochat et al. (1) proposed method of calculating the PSU prevalence, 7.61%(*n* = 74) of participants reported a high score (>5 points on the 7-point SABAS scale) on excessive smartphone use in the previous 12 months. Among them, 5.14%(*n* = 50) also gave a highly affirmative response on the conflict item of the SABAS (>6 points) and 1.64% (*n* = 16) on the unique item assessing functional impairment from smartphone use (4 points in a 1-4 scale).

The PSU mean score of female participants was statistically higher than that of male participants [*t*[2, 971] = −6.35, *p* < .001, *d* = −0.440; *M* = 4.49 [SD = 1.08] and *M* = 3.01 [SD = 1.12], respectively]. The PSU mean score of undergraduate participants was statistically higher than that of master's degree participants [*t*[2, 971] = 2.20, *p* < .0028, d = 0.171; *M* = 3.38 [SD = 1.08] and *M* = 3.19[SD = 1.20], respectively]. The PSU mean score of bisexual participants was statistically higher than that of heterosexual participants [*t*[2, 971] = −4.660, *p* < .001, *d* = −0.4227; M = 3.70 [SD = 1.02] and *M* = 3.24 [SD = 1.11], respectively].

In addition, among participants, the SUF was high than the mid-point of the scale [*M* = 3.54(SD = 1.19), 1-6 scale]. The participants' mean score in relation to an agreement that they had experienced personal, professional, or academic problems due to smartphone behavior was *M* = 1.80 (SD = .85) slightly below the mid-point of the scale (1-4-point). Overall, 421(43.27%) participants reported using smartphone health apps to improve well-being; 711(73.07%) participants reported perceived need to reduce their SUF during the previous 12 months; 705(72.46%) participants reported to have used embedded smartphone tools to control SUF during the previous 12 months.

Finally, the participants PSU total mean score was statistically correlated with their reported academic performance (grades) [*r* = −.111; 95%CI (confidence interval) [−.173, −.049], *p* < .001]. Participants with higher PSU total mean score were more likely to have relatively lower grades than those who have lower PSU total mean scores. However, it is important to emphasize that correlation does not imply causation. Given the study design, it cannot be ruled out that unmeasured or confounding variables may be driving these observed associations.

### 3.2 Significant correlates of PSU among participants (answer to RQ2)

Table 2 presents the correlations between the study's independent variables (IVs) and the dependent variable (DV). Positive correlations indicate that increases in IV values are associated with higher DV values, whereas negative correlations indicate that increases in IV values are associated with decreases in the DV.

**Table 2:** Univariate relationships between independent variables and the score of problematic smartphone use: correlation analyses' results.

Variable/measures	*r*	95% CI lower bound	95% CI upper bound	*p*
Sociodemographics variables				
Age	−.155	−.216	−.093	< .001
Gender	.202	.141	.262	< .001
Sexual orientation	.144	.082	.205	< .001
Relationship status	−.119	−.181	−.057	< .001
Education level	−.071	−.133	−.008	.028
Income	−.101	−.163	−.039	.002
Smartphone use behavior measures				
Smartphone use frequency for non-essential activities (in last 3 months; measured in hours)	.493	.444	.539	< .001
Having experienced personal, professional, or academic problems due to smartphone use (previous three months)	.424	.371	.474	< .001
Use of smartphone applications to improve well-being (previous 12 months)	.076	.013	.138	.018
Perceived need to control smartphone use frequency (previous 12 months)	.223	.162	.282	< .001
Use of embedded smartphone tools to control smartphone use frequency (previous 12 months)	.124	.061	.185	< .001
Social activities, relationships, and social engagement measures				
Number of leisure or entertainment outings with others (previous 30 days)	−.016	−.078	.047	.628
Number of close relationships (family members and friends) with whom participants engaged in leisure activities (previous 30 days)	.025	−.038	.087	.440
Satisfaction (level of) with family members and friends relationship	−.155	−.216	−.093	< .001
Satisfaction (level of) with intimate relationships	−.186	−0.246	−.125	< .001
Perceived (level of) social support from family and friends when in need	−.265	−.322	−.205	< .001
Community (level of) engagement	−.163	−.223	−.101	< .001
Religious (level of) engagement	.076	.013	.138	.017
Psychological Measures				
WMQ working memory (short-term storage) total score	.302	.244	.358	< .001
WMQ attention total mean score	.388	.333	.440	< .001
WMQ executive control total mean score	.406	.352	.457	< .001
WMQ total mean score	.439	.387	.488	< .001
UPPS-P positive urgency total mean score	.372	.316	.425	< .001
UPPS-P negative urgency total mean score	.362	.306	.415	< .001
UPPS-P urgency total mean score	.418	.365	.469	< .001
BFI extraversion total mean score	.033	−.029	.096	.297
BFI agreeableness total mean score	−.042	−.105	.021	.190
BFI consciousness total mean score	−.232	−.291	−.172	< .001
BFI neuroticism total mean score	.366	.310	.419	< .001
BFI openness total mean score	−.086	−.148	−.023	.008
Connection (level of) with future-self 20 years from the present	−.177	−.237	−.115	< .001
Mental health measures				
Sleep duration (from falling asleep until finally waking up; in last 6 months; self-estimation measured in hours)	.012	−.051	.074	.716
Sleep difficulties (difficulty falling asleep and/or frequent waking up during the night; experienced in last 6 months)	.297	.239	.354	< .001
SDHS total mean score	.332	.275	.386	< .001
SISE self-esteem	−.270	−.328	−.211	< .001
Academic Measures				
MPIL (outside classroom) total mean score	.347	.290	.401	< .001
MPIL-(inside classroom) total mean score	.245	.185	.303	< .001
Grade mean for the last university semester	−.111	−.173	−.049	< .001

*r* = correlation coefficient; CI = confidence interval; *p* = p-value (significante at *p* < 0.05).

As shown in Table 2, the vast majority of the included independent variables (IVs) were statistically correlated with the dependent variable (DV), namely participants' total mean score of problematic smartphone use (PSU). Only five of the 37 variables did not show a significant association: the number of leisure or entertainment outings with others during the previous three months; the number of family members and friends with whom participants engaged in leisure activities during the previous three months (two IVs from the social category); the total mean scores for the extraversion and agreeableness personality traits (from the psychological IV category); and sleep duration (from sleep onset to final awakening over the previous six months; from the mental health IV category).

In the sociodemographic IV category, the variables most correlated with the participants PSU total mean score (the variables with *r* > 150 and *p* < .001) were gender [*r* = .202; 95%CI (.141, .262), *p* < .001, with female participants more likely to have high PSU scores than their male counterparts] and age [*r* = −.155; 95%CI (−.216, −.093), *p* < .001]. In the smartphone use behavior IV category, the variables most correlated with the participants' SPU score were the SUF [*r* = .493; 95%CI (.444, .539), *p* < .001], having experienced personal, professional, or academic problems due to smartphone use [*r* = .424; 95%CI (.371, .474), *p* < .001], and perceived need to reduce smartphone use [*r* = .223; 95%CI (.162, .282), *p* < .001]. In the social IV category, the variables most correlated with the participants' SPU score were perceived social support from family and friends [*r* = −.265; 95%CI (−.322, −.205), *p* < .001], community engagement [*r* = −.163; 95%CI (−.223, −.101), *p* < .001], satisfaction with intimate relationships [*r* = −.186; 95% (−.246, −.125), *p* < .001], and satisfaction with family and friend relationships [*r* = −.155; 95%CI (−.216, −.093), *p* < .001]. In the psychological IV category, the variables most correlated with the participants' SPU score were cognitive difficulties related to working memory (sort-term storage, attention, and executive control) [*r* = 0.439; 95%CI (0.387, 0.488), *p* < .001], impulsivity urgency total mean score [*r* = .418; 95%CI (.365, .469), *p* < .001], neuroticism personality trait mean score [*r* = 0.366; 95%CI (.310, .419), *p* < .001], consciousness personality trait mean score [*r* = −.232; 95%CI (−.291, −.172), *p* < .001], and connection one's future-self 20 years from the present [*r* = −.177; 95%CI (−.237, −.115), *p* < .001]. In the mental health IV category, the variables most correlated with the participants' SPU score were depressive mood mean score [*r* = .332; 95%CI (.275, .386), *p* < .001], sleep difficulties [*r* = .297; 95%CI (.239, .354), *p* < .001], and self-esteem [*r* = −.270; 95%CI (−.328, −.211), *p* < .001]. In the academic IV category, the variables most correlated with the participants' PSU score were smartphone-related interference with university work (outside classroom) mean score [*r* = .347; 95%CI (.290, .401), *p* < .001] and smartphone-related interference with university work (inside classroom) mean score [*r* = .245; 95%CI (.185, .303), *p* < .001].

Finally, one important information is that all three level of difficulties on working memory dimensions were statistically correlated with the participants' academic grades: attention mean score [*r* = −.168; 95%CI (−.228, −.106), *p* < .001]; short-term storage mean score [*r* = −.108; 95%CI (−.170, −.045), *p* < .001], and executive control mean score [*r* = −.089; 95%CI (−.151, −0.026), *p* = .006]. In addition, both types of smartphone-related interference with university work were significantly correlated with the participants' academic grades: smartphone-related interference with university work (outside classroom) mean score [*r* = −.106; 95% (−.168, −.044), *p* < .001] and smartphone-related interference with university work (inside classroom) mean score [*r* = −.103; 95% (−.165, −.041), *p* = .001].

### 3.3 Significant predictors of PSU among participants (answer to RQ3)

The multivariate linear regression model showed a satisfactory coefficient of determination (R² = .50; adjusted R² = .48; RMSE = 0.790), indicating that 50% of the variance in the outcome variable was explained by variations in the predictor variables. In addition, all standard indicators commonly used to assess the quality and validity of a multivariate linear regression model were met (65–67). Specifically, the R² value attained the recommended 50% threshold, the Shapiro–Wilk normality test is non-significant (W = 0.999, *p* = .674), and inspection of the Q–Q plot indicates an approximately linear distribution of residuals around the theoretical reference line (see Supplementary Figure S1). Finally, the assessment of multicollinearity using variance inflation factors (VIF) revealed values below 2 for all predictors. Detailed information regarding the verification of the regression model assumptions is provided in the Supplementary Material.

Table 3 displays the predictive relationships between the study predictor variables (PV) and the outcome variable (OV). Positive regression coefficient (*β*) values indicate that as the value of the PV increases, the value of the OV increases as well. Negative *β* values indicate that as the value of the PV increases, the value of the OV decreases.

**Table 3:** Multivariate relationships between the independent variables and the score of problematic smartphone use: linear regression analysis' results.

Variable/measures	*β*	95% CI lower bound	95% CI upper bound	SE	*t*	*p*
Sociodemographics Variables						
Age	−0.024	−0.050	0.002	0.013	−1.780	.075
Gender						
Female—Male	−0.133	−0.285	0.018	0.077	−1.724	.085
Other—Male	−0.259	−0.806	0.288	0.278	−0.929	.353
Sexual orientation						
* *Bisexual—Heterosexual	0.182	0.029	0.335	0.078	2.335	.020
Homosexual—Heterosexual	−0.063	−0.313	0.185	0.127	−0.502	.615
Other—Heterosexual	0.229	−0.085	0.543	0.160	1.428	0.154
Relationship status	−0.016	−0.052	0.018	0.018	−0.916	.360
Education level (Master's degree—undergraduation)	0.020	−0.110	0.151	0.066	0.310	.757
Income	0.090	−0.012	0.194	0.052	1.731	.084
Smartphone use behavior measures						
Smartphone use frequency for non-essential activities (in last 3 months; measured in hours)	0.254	0.205	0.304	0.025	10.179	< .001
Having experienced personal, professional, or academic problems due to smartphone use (previous three months)	0.257	0.189	0.325	0.034	7.419	< .001
Use of smartphone applications to improve well-being (previous 12 months)	−0.114	−0.223	−0.006	0.055	−2.082	.038
Perceived need to control smartphone use frequency (previous 12 months)	0.075	−0.054	0.204	0.066	1.137	.256
Use of embedded smartphone tools to control smartphone use frequency (previous 12 months)	0.091	−0.032	0.215	0.063	1.446	.148
Social activities, relationships, and social engagement measures						
Satisfaction (level of) with family members and friends relationship	−0.086	−0.147	−0.024	0.031	−2.761	.006
Satisfaction (level of) with intimate relationships	−0.020	−0.066	0.025	0.023	−0.860	.390
Perceived (level of) social support from family and friends when in need	−0.033	−0.069	0.002	0.018	−1.842	.066
Community (level of) engagement	−0.034	−0.066	−0.002	0.016	−2.099	.036
Religious (level of) engagement	0.023	−0.008	0.055	0.016	1.4366	.151
WMQ total mean score	0.187	0.099	0.27491	0.044	4.2056	< .001
UPPS-P urgency total mean score	0.287	0.188	0.385	0.050	5.702	< .001
BFI consciousness total score	−0.071	−0.138	−0.005	0.034	−2.113	.035
BFI-10 neuroticism total score	0.076	0.018	0.13435	0.029	2.584	.010
BFI-10 openness total score	−0.031	−0.072	0.008	0.020	−1.536	.125
Connection (level of) with future-self 20 years from the present	0.007	−0.012	0.027	0.010	0.730	.465
Mental health measures						
Sleep difficulties (difficulty falling asleep and/or frequent waking up during the night; experienced in last 6 months)	0.043	−0.012	0.099	0.028	1.508	.132
SDHS total score	0.006	−0.122	0.124	0.062	0.010	.991
SISE self-esteem	−0.036	−0.110	0.036	0.037	−0.981	.327
Academic Measures						
MPIL (outside classroom) total mean score	0.041	0.007	0.074	0.016	2.426	.015
MPIL (inside classroom) total mean score	0.016	−0.015	0.047	0.016	1.020	.308
Grade mean for the last university semester	0.024	−0.033	0.082	0.029	0.824	.410

β, bêta coefficient (estimates); CI, confidence interval; SE, standard error; *t*, *t*-value; *p*, *p*-value (significance at *p* < .05).

As shown in Table 3, impulsivity (urgency impulsivity total mean score) was the strongest predictor of participants' PSU total mean score [*β* = 0.287; 95% CI (0.188, 0.385), *p* < .001]. This indicates that a one-unit increase in the impulsivity score (on a 1-4 scale) was associated with an increase of 0.287 points in the PSU score, given the positive value of the regression coefficient. Having experienced personal, professional, or academic problems due to smartphone use behavior during the previous three months was the second most important predictor of the participants' PSU score [*β* = 0.257; 95%CI (0.189, 0.325), *p* < .001]. The SUF for non-essential activities during the three previous months was the third strongest predictor of the participants' PSU score [*β* = 0.254; 95%CI (0.205, 0.304), *p* < .001]. The overall score of cognitive difficulties related to working memory was the fourth most important predictor of the participants' SPU score [*β* = 0.187; 95%CI (0.099, 0.274), *p* < .001]. The sexual orientation (bisexual vs. heterosexual) was the fifth strongest predictor of the participants' PSU score [*β* = 0.182; 95%CI (0.029, 0.335), *p* = .020]. To have used smartphone apps to improve well-being (in the last 12 months) was the sixth most important predictor of the participants SPU score [*β* = −0.114; 95%CI (−0.223, −0.006), *p* = .038].

Other relatively weak but statistical significant and negative predictors of the participants' SPU score included: satisfaction with family and friend relationships; community engagement; and consciousness personality trait. An increase on the values of these predictors decreases the value of the outcome variable. Relatively weak but significant and positive predictors of the participants' PSU score included: neuroticism personality trait and smartphone-related interference with university work (outside classroom). Conversely, an increase on the values of these predictors increases the value of the outcome variable. See Table 3 for the detailed predictive statistics related to the above described relationships.

### 3.4 Significant moderators and mediators between SUF and PSU among participants (answer to RQ4)

As a reminder, we examined the following variables as potential moderators of the association between smartphone use frequency (SUF) and problematic smartphone use (PSU): satisfaction with family and friends' relationships, satisfaction with intimate relationships, perceived social support, community engagement, religious engagement, and connection with the future-self. Only 2 of these 6 variables were significant and positive moderators: community engagement and connection with future-self. Indeed, the interaction between SUF and community engagement was found to be statistically significant [*β* = 0.037, 95%CI (0.004, 0.068), *p* = .020]. At low moderation SUF = 0.383, 95% CI (0.309, 0.459), *p* < .001. At middle moderation SUF = 0.447, 95% CI (0.392, 0.500), *p* < .001. At high moderation SUF = 0.510, 95% CI (0.431, 0.588), *p* < .001. The interaction between SUF and connection with future-self was found to be statistically significant [*β* = 0.015, 95%CI (0.001.85, 0.029), *p* = .030]. At low moderation SUF = 0.173, 95% CI (0.114, 0.233), *p* < .001. At middle moderation SUF = 0.218, 95% CI (0.180, 0.261), *p* < .001. At high moderation SUF = 0.263, 95% CI (0.211, 0.319), *p* < .001.

It must be noted that, in a moderation analysis, a positive beta coefficient for the interaction term indicates that as the moderator increases, the relationship between the predictor and the outcome becomes more positive (or less negative).

We tested the following variables as mediators: sleep difficulties, happiness-depressive mood, and self-esteem. The indirect effects of SUF on SPU was found to be statistically significant with all these three mediators, respectively: effect = 0.043, 95% CI (0.027, 0.060), *p* < .001, mediation % = 9.43; effect = 0.047, 95% CI (0.029, 0.065), *p* < .001, mediation % = 10.3; effect = 0.0294, 95% CI (0.014, 0.045), *p* < .001, mediation % = 6.39. These results support partial mediation. However, it must be noted that the testing design does not infer causality.

## 4 Discussion

The present study investigated the prevalence, correlates, predictors, and underlying mechanisms of problematic smartphone use (PSU) among French psychology university students using an integrated analytical framework. Overall, the findings align closely with contemporary theoretical models conceptualizing PSU as a multifactorial phenomenon driven by behavioral exposure, psychological vulnerabilities, and contextual factors (3–5). Importantly, the results also highlight specificities of psychology students, a population theoretically more aware of addiction mechanisms, yet still substantially affected by PSU.

### 4.1 Level and prevalence of PSU among psychology students

The mean PSU score observed in this sample (M = 3.34 on a 1–7 scale) was slightly below the scale midpoint, suggesting moderate overall levels of PSU. However, distributional analyses revealed that a substantial proportion of students reported elevated levels: using a conservative estimation method (1), the prevalence of PSU is between 1.64% and 5.14%. However, according to an alternative prevalence estimation method (49), 25% of participants exceeded the cut-off score of 4.14. The latter prevalence estimate was consistent with previous European and French studies reporting PSU rates between 20% and 35% among university students (11–13).

Contrary to a potential “protective knowledge” hypothesis, psychology students did not appear less affected than other student populations. This finding supports the notion of an intention–behavior gap (41): awareness of addiction risks does not necessarily translate into healthier smartphone use behaviors. One may hypothesize that smartphones may still function as readily accessible tools for coping with stress, emotional demands, and academic pressure, even among future mental health professionals (5, 42).

### 4.2 Sociodemographic correlates and hypothesis testing

Most sociodemographic hypotheses were confirmed. Younger age, female gender, bisexual orientation, and undergraduate status were all associated with higher PSU, in line with previous literature (11, 16, 17, 19). The stronger PSU observed among women may reflect greater reliance on smartphones for social interaction and emotion regulation (18). Higher PSU among students with bisexual orientation aligns with studies suggesting that sexual minority individuals may rely more heavily on digital environments, possibly, for social support and identity affirmation (19).

Income and relationship status showed weaker or inconsistent associations once other variables were controlled, suggesting that their effects may be indirect or mediated by social and psychological resources rather than exerting strong independent influences. Overall, sociodemographic factors played a role, but their predictive power was modest compared to behavioral and psychological variables.

### 4.3 Smartphone use behaviors as proximal determinants

Consistent with prior research, smartphone use frequency (SUF) for non-essential activities emerged as one of the strongest correlates and predictors of PSU (1, 12). Experiencing problems related to smartphone use was an even stronger predictor, supporting the conceptualization of PSU as a pattern of use associated with functional impairment rather than mere high engagement (1).

Interestingly, the use of well-being apps was negatively associated with PSU in the multivariate model, contrary to the initial hypothesis. This finding suggests that, after accounting for other risk factors, engagement with such apps may be indicative of greater awareness of wellbeing and mental health needs as well as more proactive self-regulatory strategies among some students (68). However, the bivariate associations and existing literature also indicate that perceived need to reduce smartphone use and reliance on control tools can signal underlying dysregulation (21, 22). These mixed findings underline the importance of distinguishing between adaptive and maladaptive self-regulation strategies.

Overall, the results delineate two related regulatory pathways, whereby well-being apps are associated with adaptive, need-driven self-regulation, while control-oriented smartphone tools may index compensatory responses to perceived dysregulation.

### 4.4 Social variables: partial support and unexpected moderation effects

As hypothesized, several social variables—particularly social support, relationship satisfaction, and community engagement—were negatively correlated with PSU, supporting compensatory models of technology use (42, 69). Students with stronger offline social resources appear less prone to excessive or dysregulated smartphone use.

However, moderation analyses yielded unexpected results. Community engagement and connection with one's future-self positively moderated the SUF–PSU relationship, meaning that at higher levels of these variables, the association between SUF and PSU was stronger rather than weaker. This finding contradicts the initial hypothesis that these factors would buffer against PSU and stands in contrast to the observed direct associations between the two predictor variables and participants' PSU scores. Specifically, higher levels of community engagement and stronger connection with the future self were significantly associated with lower PSU scores. One possible explanation is that socially and future-oriented students may use smartphones more intensively to coordinate activities, maintain commitments, or pursue long-term goals, thereby amplifying the translation of high use into problematic patterns. Alternatively, these findings may reflect complex interactions between high engagement, role overload, digital dependence, and digital well-being (70) in highly active students. Such results suggest that social and motivational resources are not universally protective and may, under certain conditions, exacerbate PSU risk. In future studies, we recommend the adoption of mixed-methods designs combining interview-based and questionnaire-based approaches. Such designs would be better suited to exploring in greater depth the complex roles—particularly moderation and mediation—played by social factors, including satisfaction with social relationships, community engagement, and religiosity, in the relationships between SUF and PSU.

Regarding this mediation and moderation analyses, it is important to emphasize that the cross-sectional nature of the data precludes the establishment of temporal precedence and, consequently, does not allow for direct inferences regarding causal mechanisms (71, 72). Alternative directional relationships and reciprocal influences among the studied variables therefore remain plausible. Accordingly, the mediation pathways identified in the present study should be interpreted as statistical indirect associations rather than evidence of causal processes.

Nevertheless, the tested models were derived from established theoretical frameworks and informed by prior empirical findings reviewed in the introduction. As such, the observed mediation and moderation effects provide preliminary support for theoretically grounded relationships among the variables under investigation.

### 4.5 Psychological and mental health vulnerabilities

Psychological variables emerged as central determinants of PSU. Impulsivity was the strongest predictor in the multivariate model, confirming a robust body of evidence linking urgency-driven impulsivity to addictive and compulsive behaviors (26–28). Working memory dysfunction was also a strong predictor, supporting prior findings that attentional and executive control deficits increase susceptibility to distraction and compulsive smartphone checking (23, 25).

Personality hypotheses were largely confirmed: conscientiousness was negatively associated with PSU, while neuroticism showed a positive association. This is in line with previous studies (29). Connection with the future-self was negatively correlated with PSU, consistent with theories emphasizing the role of temporal self-continuity in self-regulation and addictive behaviors (28, 29), and with other studies on personality and addictive behaviors (73).

Mental health variables partially mediated the relationship between SUF and PSU. Sleep difficulties, depressive mood, and low self-esteem each accounted for a modest but significant proportion of the association, supporting bidirectional and self-reinforcing models in which heavy smartphone use contributes to psychological distress, which in turn exacerbates PSU (33, 35, 36).

Together, these findings contribute to the existing literature by identifying patterns of association that are consistent with current conceptual models of problematic smartphone use, namely the Interaction of Person–Affect–Cognition–Execution (I-PACE) model [4, 7) and the compensatory internet use theories (42).

### 4.6 Academic functioning and PSU

Consistent with previous studies, higher PSU was associated with greater smartphone-related interference with academic work and with lower academic grades (38–40). Although effect sizes were small, these findings reinforce concerns about the cumulative impact of multitasking and attentional disruption on learning outcomes.

The observed associations between working memory difficulties, smartphone interference, and grades further suggest that cognitive vulnerabilities may be a key mechanism linking PSU to academic underperformance. However, the design of the present study does not allow us to disentangle the extent to which participants' academic performance is negatively affected by working memory difficulties, nor to determine to what extent these difficulties are attributable to patterns of smartphone use or to other unmeasured factors. Indeed, prospective, cluster-based longitudinal studies are needed to identify distinct patterns of association between cognitive vulnerabilities, problematic smartphone use, and academic functioning, and to clarify how these profiles evolve over time and contribute to academic underperformance and/or the maintenance of problematic smartphone use.

### 4.7 The explained variance

In the multivariate regression model, the included predictors accounted for only 50% of the variance in the outcome variable. This indicates that a substantial proportion of the variance in PSU scores remains unexplained and is likely attributable to additional factors not included in the model. In particular, part of this unexplained variance may be related to the nature of online interactions and individuals' subjective experiences of these interactions.

## 5 Limitations and recommendations for future studies

Several limitations of the present study should be acknowledged.

First, an important limitation concerns the assessment of problematic smartphone use (PSU). Although the present study employed the Smartphone Application-Based Addiction Scale (SABAS) (45), which is grounded in the components model of addiction and has demonstrated satisfactory psychometric properties across multiple cultural contexts, considerable debate remains regarding the conceptualization and measurement of PSU. Different assessment instruments are based on distinct theoretical frameworks and often rely on different scoring procedures and cut-off criteria, resulting in substantial variability in prevalence estimates (3, 43, 44). Consequently, the prevalence rates reported in this study should be interpreted with caution and considered within the context of the specific measurement approach adopted. Future research would benefit from greater consensus regarding the definition and assessment of PSU, as well as from studies directly comparing multiple instruments within the same population.

Second, the focus on PSU as a global construct may have limited the ability to distinguish among specific smartphone-mediated activities that could be differentially associated with cognitive, psychological, and academic outcomes. Future research would benefit from adopting more fine-grained, activity-specific approaches, as well as broader human–digital service interaction perspectives, to better disentangle the underlying mechanisms linking smartphone use to academic functioning and digital well-being.

Third, the cross-sectional design precludes causal inference and does not allow conclusions regarding the temporal ordering of the observed associations. Consequently, reciprocal and bidirectional relationships among PSU, mental health, and academic outcomes remain plausible. Future studies should employ longitudinal, prospective, and cross-lagged designs to clarify temporal dynamics and address potential endogeneity among these variables.

Fourth, all measures relied on self-report data, which may be susceptible to recall errors and social desirability bias. In particular, the absence of objective smartphone-use indicators (e.g., screen-time records, application usage logs, or passive sensing data) represents an important limitation. Future studies should combine validated self-report instruments, such as the SABAS, with objective behavioral measures obtained directly from smartphones. Such multimethod approaches would provide a more comprehensive assessment of both subjective experiences of problematic use and actual patterns of smartphone engagement.

Fifth, several constructs, including social variables, future-self connection, and self-esteem, were assessed using single-item measures. Although single-item indicators may be appropriate in large-scale surveys when questionnaire length is a concern, they may provide lower reliability and reduced sensitivity compared to multi-item scales. In addition, some personality dimensions assessed with the BFI-10 exhibited relatively low internal consistency, a limitation commonly associated with very brief personality inventories.

Finally, data were collected from only two French universities, which can be considered as limitation. However, approximately 75% of the target population within these institutions completed the survey. Furthermore, French university students tend to share relatively similar sociodemographic characteristics across institutions (74, 75). Nevertheless, the extent to which the present sample is representative of the broader population of French university students remains uncertain. Accordingly, the generalizability of the findings should be considered with caution and requires replication in more diverse and nationally representative samples.

## 6 Conclusion

This study provides a comprehensive examination of PSU among French psychology students and demonstrates that substantial levels of PSU persist despite high theoretical awareness of addiction mechanisms. The findings confirm the central role of impulsivity, cognitive control difficulties, intensive smartphone use, and mental health vulnerabilities, while also revealing complex and sometimes counterintuitive roles of social and motivational factors.

Together, these results underscore the need for prevention and intervention strategies that go beyond awareness-raising and instead target self-regulation skills, emotional coping, and academic context–specific smartphone use. Tailored interventions within psychology curricula may be particularly relevant, both to improve students' well-being and to prepare future mental health professionals to address technology-related addictions in others.

## Data Availability

The raw data supporting the conclusions of this article will be made available by the authors, without undue reservation.
