# Supplementary material for: Demographic, social, psychological, mental health, and academic correlates of problematic smartphone use among French psychology students
Source: Front Digit Health. 2026 Jul 3;8:1784927. doi: 10.3389/fdgth.2026.1784927 (PMC13375732; doi:10.3389/fdgth.2026.1784927)

Supplementary Material

Table S1. Normality Test

Normality Test (Shapiro-Wilk)

Statistic	p
0.999	0.674

Table 2. Multicollinearity statistics

Collinearity Statistics

	VIF	Tolerance
age	1.16	0.861
gender	1.22	0.822
sex_orientation	1.07	0.938
relationship_status	1.24	0.808
edu_level	1.07	0.933
income	1.11	0.900
smartphone_use_time	1.16	0.861
problem_because_smartphoneUse	1.16	0.864
use_apps_wellbeing	1.06	0.943
need_smartphone_use reduction	1.14	0.879
use_tools_smartphone_control	1.10	0.907
satisfaction_relation_closePeople	1.17	0.856
satisfaction_intimate_relationship	1.24	0.803
social_support_familly_friends	1.20	0.831
community_engagement	1.08	0.929
religiosity_engagement	1.11	0.902
mem_score	1.31	0.766
attention_score	1.42	0.704
executive_score	1.42	0.705
UPPS_positive_score	1.28	0.780

Collinearity Statistics

	VIF	Tolerance
UPPS_negative_score	1.24	0.804
consc_score	1.31	0.763
neuro_score	1.50	0.666
openess_score	1.04	0.959
future_self_10years	1.15	0.871
SHI_sleep_difficulties	1.23	0.810
SDHS_score	1.41	0.710
SISE_self_esteem	1.28	0.780
MPIL_outClass_score	1.54	0.650
MPIL_inClass_score	1.37	0.731
grade_category	1.13	0.885

Figure S1. Q-Q Plot

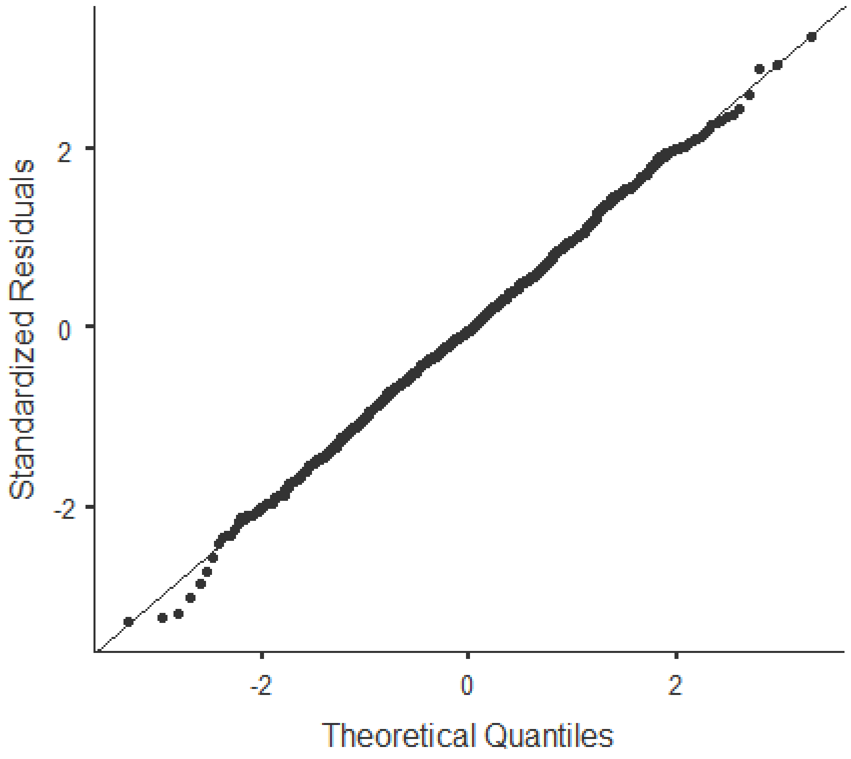

Supplement: Supplementary file 1 [file Datasheet1.pdf]
